# Supplementary material for: The Neonatal Fc Receptor Is Elevated in Monocyte-Derived Immune Cells in Pancreatic Cancer
Source: Int J Mol Sci. 2022 Jun 25;23(13):7066. doi: 10.3390/ijms23137066 (PMC9266939; doi:10.3390/ijms23137066)
Supplement: Supplementary file 1 [file ijms-23-07066-s001.zip › ijms-1744541-supplementary.pdf]

Supplemental Figures

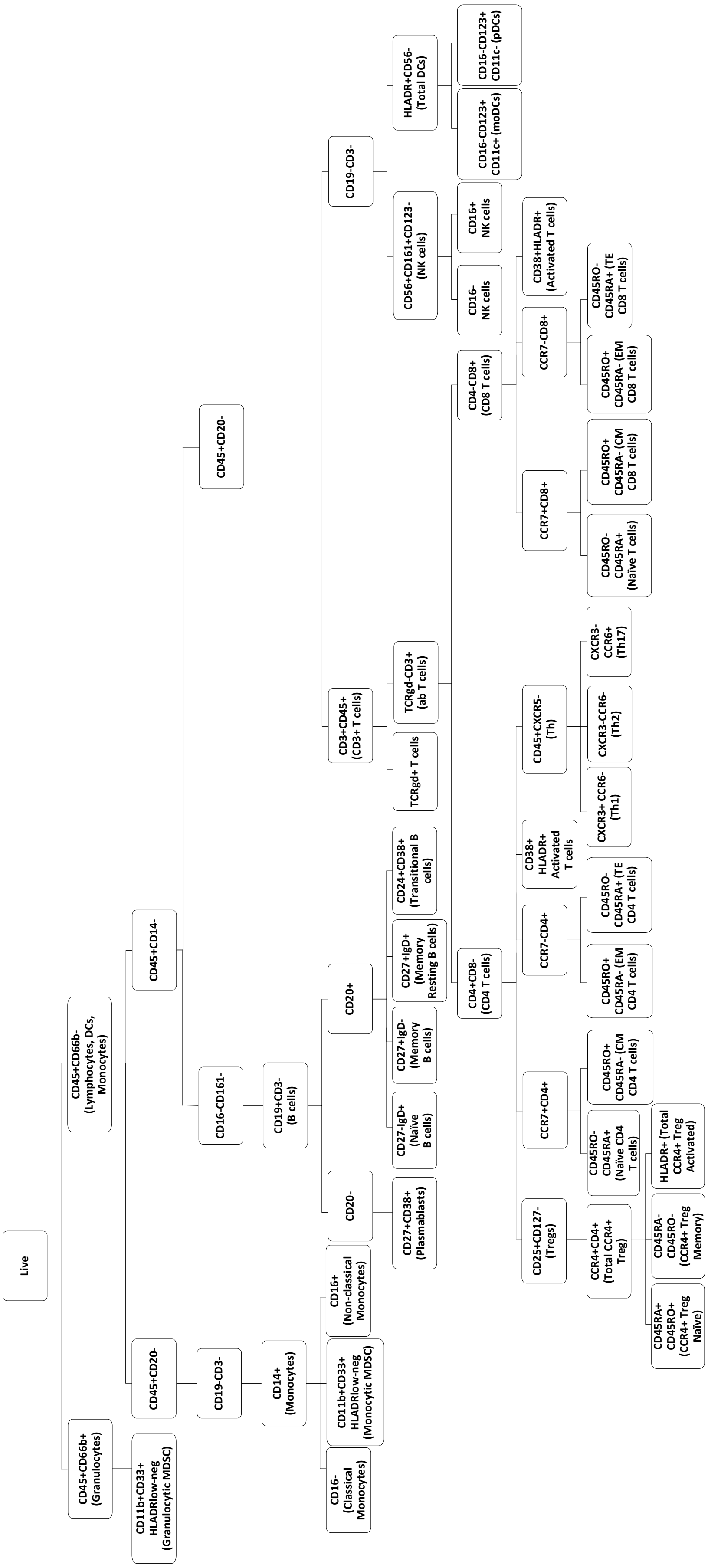

Supplemental Figure S1. Gating Strategy for CyTOF Populations. The flowchart for the CyTOF gating is shown. Boxes represent the populations and arrows represent the gates within those populations.

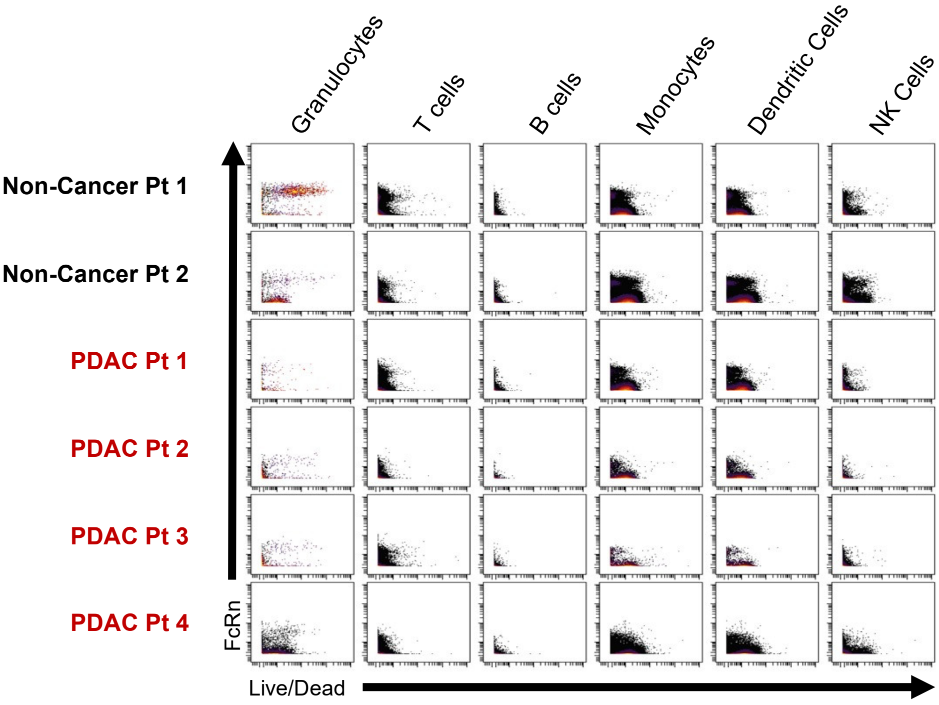

Supplemental Figure S2. Representative Dotplots of FcRn. Representative FcRn dotplots of two non-cancer and four PDAC samples across the six main immune populations (granulocytes, T cells, B cells, monocytes, dendritic cells, NK cells).

Supplemental Tables

Supplemental Table S1. Metal Isotope-bound Antibodies. A list of all metal isotope-bound antibodies used in CyTOF staining.

Marker	Metal	Marker	Metal	Marker	Metal	Marker	Metal
CD196/CCR6	141Pr	CD161	151Eu	CD56	163Dy	IgD	174Yb
CD123/IL3R	143Nd	CD194/CCR4	152Sm	TCRy	164Dy	CD127	176Yb
CD19	144Nd	CD25	153Eu	CD294	166Er	CD45	89Y
CD4	145Nd	CD27	154Sm	CD197/CCR7	167Er	Viability	103Rh
CD8a	146Nd	CD57	155Gd	CD14	168Er	CD279	175Lu
CD11c	147Sm	CD183/CXCR3	156Gd	CD3	170Er	CD11b	209Bi
CD16	148Nd	CD185/CXCR5	158Gd	CD20	171Yb	CD33	165Ho
CD45RO	149Sm	CD28	160Gd	CD66b	172Yb	CD200	162Dy
CD45RA	150Nd	CD38	161Dy	HLA-DR	173Yb	FcRn	169Tm

Supplemental Table S2. Gating Strategy for Murine Splenocyte Immune Populations. Nine different immune populations were stained using the gating strategies outlined.

Immune Population	Gating
Monocytic DC	MHC class $II^{+}CD11b^{+}CD11c^{+/-}Ly6C^{+}DC\text{-}sign^{+}$
Langerhans DC	MHC class $II^{+}CD11b^{+}CD11c^{+}Ly6C^{-}DC\text{-}sign^{+}$
Classical DC1	$CD8a^{+}CD11b^{-}CD24^{++}$
Classical DC2	$CD11b^{+}CD103^{-}CD8a^{-}CD24^{+/-}$
Migratory cDC2	$CD11b^{+}CD103^{+}CD8a^{-}CD24^{++}$
Plasmacytoid DC	$CD11b^{-}CD11c^{+}Ly6C^{+}CD317^{+}$
Macrophage	$CD11b^{+}CD64^{+}F4/80^{+}$
Monocytic MDSC	$CD11b^{+}Ly6C^{+}Ly6G^{-}$
Granulocytic MDSC	$CD11b^{+}Ly6C^{+/-}Ly6G^{+}$

Supplemental Table S3. Gating Strategy for Human PBMC Immune Populations. Eight of the gating strategies for different relevant immune populations.

Immune Population	Gating
Granulocytes	$CD45^{+}CD66b^{+}$
T Cells	$CD45^{+}CD66b^{-}CD14^{-}CD20^{-}CD3^{+}$
B Cells	$CD45^{+}CD66b^{-}CD14^{-}CD16^{-}CD161^{-}CD19^{+}CD3^{-}$
Natural Killer Cells	$CD45^{+}CD66b^{-}CD20^{-}CD19^{-}CD3^{-}CD56^{+}CD161^{+}CD123^{-}$
Monocytes	$CD45^{+}CD66b^{-}CD20^{-}CD19^{-}CD3^{-}CD14^{+}$
Dendritic Cells	$CD45^{+}CD66b^{-}CD20^{-}CD19^{-}CD3^{-}CD14^{+}HLADR^{+}CD56^{-}$
Monocytic MDSC	$CD45^{+}CD66b^{-}CD20^{-}CD19^{-}CD3^{-}CD14^{+}CD11b^{+}CD33^{+}HLADR^{low/neg}$
Granulocytic MDSC	$CD45^{+}CD66b^{+}CD11b^{+}CD33^{+}HLADR^{low/neg}$
